# Proteomic analysis of decellularized mice liver and kidney extracellular matrices

**DOI:** 10.1186/s13036-024-00413-8

**Published:** 2024-02-22

**Authors:** Anna-Maria Diedrich, Assal Daneshgar, Peter Tang, Oliver Klein, Annika Mohr, Olachi A. Onwuegbuchulam, Sabine von Rueden, Kerstin Menck, Annalen Bleckmann, Mazen A. Juratli, Felix Becker, Igor M. Sauer, Karl H. Hillebrandt, Andreas Pascher, Benjamin Struecker

**Affiliations:** 1 Department of General, Visceral, and Transplant Surgery, University Hospital Muenster, 48149 Muenster, Germany; 2 Department of Surgery, Charité Mitte | Campus Virchow-Klinikum, Charité -Universitaetsmedizin Berlin, Campus, 13353 Berlin, Germany; 3 Berlin Institute of Health at Charité – Universitaetsmedizin Berlin, Core Facility Imaging Mass Spectrometry, 13353 Berlin, Germany; 4 Department of Medicine A for Hematology, Oncology, Hemostaseology and Pneumology, University Hospital Muenster, 48149 Muenster, Germany; 5 West German Cancer Center, University Hospital Muenster, 48149 Muenster, Germany; 6 Berlin Institute of Health at Charité – Universitaetsmedizin Berlin, BIH Biomedical Innovation Academy, BIH Charité Clinician Scientist Program, Charitéplatz 1, 10117 Berlin, Germany

**Keywords:** Decellularized scaffolds, Mice liver/kidney matrisome, Bottom-up proteomics, Tissue engineering

## Abstract

**Background:**

The extracellular matrix (ECM) is a three-dimensional network of proteins that encases and supports cells within a tissue and promotes physiological and pathological cellular differentiation and functionality. Understanding the complex composition of the ECM is essential to decrypt physiological processes as well as pathogenesis. In this context, the method of decellularization is a useful technique to eliminate cellular components from tissues while preserving the majority of the structural and functional integrity of the ECM.

**Results:**

In this study, we employed a bottom-up proteomic approach to elucidate the intricate network of proteins in the decellularized extracellular matrices of murine liver and kidney tissues. This approach involved the use of a novel, perfusion-based decellularization protocol to generate acellular whole organ scaffolds. Proteomic analysis of decellularized mice liver and kidney ECM scaffolds revealed tissue-specific differences in matrisome composition, while we found a predominantly stable composition of the core matrisome, consisting of collagens, glycoproteins, and proteoglycans. Liver matrisome analysis revealed unique proteins such as collagen type VI alpha-6, fibrillin-2 or biglycan. In the kidney, specific ECM-regulators such as cathepsin z were detected.

**Conclusion:**

The identification of distinct proteomic signatures provides insights into how different matrisome compositions might influence the biological properties of distinct tissues. This experimental workflow will help to further elucidate the proteomic landscape of decellularized extracellular matrix scaffolds of mice in order to decipher complex cell–matrix interactions and their contribution to a tissue-specific microenvironment.

**Supplementary Information:**

The online version contains supplementary material available at 10.1186/s13036-024-00413-8.

## Introduction

In biological tissues, cells are embedded within a complex, three-dimensional extracellular matrix (ECM) mediating crucial biomechanical and biochemical processes. This reservoir of macromolecules is vastly determined by the presence of highly crosslinked proteins and an extensive number of associated regulators and factors, which together are widely referred to as the matrisome [1, 2]. The matrisome contributes to the spatial arrangement of cells and the formation of entire tissues by its key influence on vital processes in tissue homeostasis and regeneration [3, 4]. The continuous and dynamic interaction between cells and their surrounding ECM, referred to as cell-ECM dynamic reciprocity, is the key driver of many biological processes, including maintaining structural integrity, regulating tissue regeneration, and mediating signaling pathways [5]. Furthermore, the ECM plays a pivotal role in the development of pathological conditions. Understanding its intricate structure has gained increasing significance in the study of physical tissue properties based on cell–matrix interactions and holds implications for advancing regenerative medicine [6].

In this context, the process of tissue or organ decellularization serves as a valuable technique: This method effectively eliminates cellular components from tissues while largely preserving the structural and functional integrity of the ECM. These naturally tissue-derived biological scaffolds maintain the native architecture and may demonstrate superiority over alternative scaffolds e.g., based on Matrigel™, a reconstituted basement membrane matrix derived from extracts of Engelbreth-Holm-Swarm mouse tumors [7]. Decellularized extracellular matrices (dECM) offer a unique platform for investigating intricate cell–matrix interactions. Two methods are used to generate dECM: cell-derived dECM scaffolds are created by cell-specific secretion of cultured cells in vitro, and tissue/organ-derived dECM scaffolds are created by perfusion or immersion of tissue slices, parts, organs as a whole, or after homogenization [8, 9]. Decellularizing a whole organ has the advantage of preserving the original anatomical structure of the ECM, the spatial arrangement of matrisome proteins, and the ECM integrity, which allows a more accurate representation of in vivo conditions.

In addition, physical methods of decellularization including mechanical forces and temperature-based techniques [10], can be differentiated from chemical methods through the use of detergents, and from enzymatic methods employing enzymes such as collagenases, lipases, trypsin, and nucleases. Combining different techniques can enhance the tissue decellularization process [11]. Recently, novel decellularization methods, such as vacuum-assisted decellularization and apoptosis-assisted decellularization have emerged as promising alternatives to traditional approaches. While these methods show evidence of strong efficacy, they are not yet widely studied and implemented [12, 13]. To evaluate and compare the biological activity of dECM-based materials, decellularized scaffolds have undergone thorough characterization of both their biochemical and biophysical properties to identify key components that may be of particular importance in interpreting cellular response [14]. Thus, the current efforts aim to further unravel the complex interactions between extracellular matrices and their host cells to provide in-depth information relevant for the interpretation of bioactivity [15]. Analyzing ECM proteins in depth remains a challenge because many ECM proteins are insoluble and extensively cross-linked [16]. Therefore bottom-up proteomic methods have become an attractive method for determining the proteomic composition of bioengineered tissue models via proteolytic digestion of protein extracts and subsequent mass spectrometry analysis [17, 18]. Bottom-up proteomics performed on cell or tissue lysates, also known as shotgun proteomics, enables highly sensitive detection and presents a powerful approach for obtaining robust information on complex protein mixtures [19, 20].

In this study, we provide comprehensive new data on the matrisome of decellularized extracellular matrices of mouse liver and kidney obtained through shotgun proteomics. This effort aims to enhance our understanding of the proteomic distinctions within these natural matrices. By employing whole organ perfusion decellularization, this work presents a novel experimental workflow to effectively produce acellular mouse liver and kidney extracellular matrix scaffolds that can serve as naturally derived platforms to investigate proteomic ECM composition in health and disease.

In summary, the integration of decellularization techniques and proteomic analysis is an innovative and powerful toolkit for unraveling the intricate molecular networks of extracellular matrices.

## Material and methods

### Animals

For animal experiments twenty C57BL/6 wildtype mice (Charles River Laboratories, Sulzfeld, Germany) were utilized and housed at the local animal facility (ZTE, Universitaetsklinikum Muenster, Muenster, Germany). The care and handling of animals as well as all protocols and procedures were approved by the Regional Veterinary Office and the State Agency for Nature, Environment and Consumer Protection, North Rhine-Westphalia, Germany (LANUV – Landesamt fuer Natur, Umwelt und Verbraucherschutz, File No. 81–02.04.2020.A423). Organ procurement under sterile conditions for decellularization was performed on ten male mice (6–9 weeks old, 20–30 g), resulting in ten livers and eight kidneys available for decellularization. Additionally, we employed another ten mice to obtain native controls without subsequent decellularization. These specimens were stored at -80ºC for further analysis.

### Mouse liver and kidney harvesting

The organ procurement procedure was adapted from a method described by *Hillebrandt *et al*.* [21] for rat liver explantation. The procedure was carried out under inhalative isoflurane anesthesia (Forene®, AbbVie, Wiesbaden, Germany) and a weight-adjusted subcutaneous buprenorphine injection 30 min prior to surgery for analgesia (0.1 mg/kg bodyweight, buprenorphine hydrochloride, Indivior, North Chesterfield, USA). In brief, after shaving and disinfecting the abdomen, a median laparotomy was performed followed by preparation of the liver. Afterwards, the bile duct and side branches of the celiac trunk were clipped and dissected to further mobilize and prepare the portal vein for cannulation. Subsequently, an incision into the portal vein was made. An obliquely shortened 24G catheter (BD Insyte-W 24, Becton Dickinson AG, Basel, Switzerland) was introduced into the vessel under slow running of 50 ml 0.9% saline infusion (B. Braun, Melsungen, Germany) with an additive of 1,000 Units heparin (Ratiopharm, Ulm, Germany). Under saline perfusion and visual control, the liver inflated and started to decolorize. To allow outflow of the perfusate, an incision was made into the inferior vena cava. Ultimately, exsanguination led to the animal’s death which was confirmed by the absence of a heartbeat. The cannula was then fixed inside the portal vein with 7–0 sutures (Resorba, Nuernberg, Germany) and metal clips (Peters Surgical, Boulogne-Billancourt, France). To explant the whole liver, the surrounding connective tissue was dissected, and the attached ligaments were removed.

For the removal of the kidneys the distal part of the aorta was searched in the retroperitoneal space right above the bifurcation into the iliac arteries. A 26G catheter (Abbocath™-T 26G, ICU Medical, San Clemente, CA, USA) was inserted into the distal aorta while the suprarenal aorta and the superior mesenteric artery were ligated tightly. Using a syringe, 10 ml of 0.9% saline solution was injected through the cannula so that both kidneys simultaneously inflated and decolorized. The renal veins and the opened inferior vena cava served as perfusate outflow. Finally, the cannula was fixed inside the vessel and both kidneys (connected via the aorta) were explanted.

### Liver decellularization via portal venous perfusion

The setup and protocol for liver decellularization was modified from *Struecker *et al*.* [22]*.* For perfusion decellularization a roller pump (Reglo ICC Digital Peristaltic Pump, Ismatec, Switzerland) was utilized. The liver was first perfused with phosphate buffered saline (PBS, VWR International, Darmstadt, Germany) for 10 min, then for 90 min with 1% Triton X-100 and 90 min with 1% sodium dodecyl sulfate (SDS) (both from Carl Roth, Karlsruhe, Germany) followed by another 10 min of perfusion with PBS. During the decellularization process the roller pump was set to a constant flow rate of 3.8 ml/min. After decellularization each liver was divided into three parts: the lateral lobe was fixed in 4% formalin (Langenbrinck, Emmendingen, Germany) for histological analysis, the medial lobe was stored at -80°C for proteomics and the right and caudal lobes were stored at -80°C for biochemical analysis and DNA quantification.

### Kidney decellularization via distal aortic perfusion

Decellularization of a pair of kidneys was performed employing the same instrumental set up as for liver decellularization. For kidney decellularization, the roller pump was set to a flow rate of 2.4 ml/min. The kidneys were first perfused with PBS for 10 min, followed by 30 min of perfusion using 1% Triton X-100 and 120 min using 1% SDS. Finally, the kidneys were perfused for 10 min with PBS. Each kidney was then divided into three parts: one part for histological analysis (fixed in 4% formalin), another part for proteomics (stored at -80°C), and a third part for biochemical analysis including DNA quantification (stored at -80°C).

### Histological analysis and immunohistochemistry of dECMs

For histological analysis, the tissue samples were fixed in 4% formalin for 24–48 h, then dehydrated and embedded in paraffin (Merck Chemicals GmbH, Darmstadt, Germany). The paraffin blocks were cut non-consecutively into 3 µm thin slices by using a microtome (Microm HM 360, Carl Zeiss, Oberkochen, Germany).

To assess the success of decellularization, Hematoxylin (AppliChem, Darmstadt, Germany) and Eosin (Morphisto, Offenbach am Main, Germany) staining was performed. The dECM scaffolds were further evaluated by performing Elastica-van-Gieson (Morphisto #12,739), Alcianblue-PAS (Morphisto #11,388) and Picro-Siriusred Collagen I&III (Morphisto #13,425) staining according to the manufacturer’s instructions.

For immunohistochemical staining, paraffin sections were deparaffinized and rehydrated. Antigen retrieval was performed using 10 mM citrate buffer (pH 6.0, Santa-Cruz Biotechnology, Dallas, Texas, USA) followed by overnight incubation at 4°C with the primary antibodies: rabbit polyclonal anti-laminin antibody 1:30 (#ab11575, Abcam, Cambridge, United Kingdom) and rabbit polyclonal anti-collagen IV antibody 1:200 (#ab19808, Abcam). As a secondary antibody, a 1:25 diluted goat anti-rabbit HRP IgG antibody (Sigma-Aldrich, St. Louis, MO, USA, Cat 6154) was used and incubated for one hour at room temperature. Visualization was then performed with DAB solution (Santa Cruz, sc-24982) followed by counterstaining with Gills Hematoxylin (Santa Cruz, sc-24973). Sections were then mounted in Organo/Limonene mount (Santa Cruz, sc-45087). After conducting proteomic analysis, we performed additional immunohistochemical staining to confirm organ specific proteins using the protocol described above with DAB visualization. For liver tissues we employed a rabbit polyclonal biglycan antibody diluted to 1:20 (16,409–1-AP, proteintech®, Rosemont, IL, USA) as primary antibody and 1:200 diluted goat anti-rabbit HRP IgG antibody (#ab6721, Abcam) as secondary antibody. For kidney tissues, we utilized a 1:75 dilution of goat polyclonal anti-cathepsin Z antibody (#AF934, R&D Systems, Minneapolis, USA) as primary antibody and a 1:200 dilution of rabbit anti-goat HRP IgG antibody (Cat A5420, Sigma Aldrich) as secondary antibody.

DAB visualization enables high specificity in staining and differentiation between specific and non-specific staining. We also implemented immunofluorescence visualization, as it offers very high sensitivity and allows the detection of small amounts of a target antigen. Overall, the use of both methods enables a more comprehensive and detailed characterization of protein expression in tissues.

For this purpose, another set of deparaffinized and rehydrated tissue sections were incubated after being subjected to the primary antibodies for laminin and collagen IV with a goat anti-rat FITC IgG2a secondary antibody at a dilution of 1:500 (Novus Biologicals LLC, Centennial, CO, USA, NB7124) together with 4’,6-diamidino-2-phenylindol (DAPI #62,248, Thermo Fisher Scientific, Waltham, MA USA) for one hour at room temperature. Sections were then mounted in Immu-Mount (Shandon ™Immu-Mount™, Thermo Fisher Scientific).

All microscopic images were obtained using a BZ-X800 Keyence fluorescence microscope (Keyence Deutschland GmbH, Neu-Isenburg, Germany).

### DNA isolation and quantification

DNA content was isolated and quantified after decellularization using the DNeasy Blood & Tissue Kit (Qiagen, Hilden, Germany) and the Qiagen QIAcube. The dECM scaffolds were stored at -80ºC until being thawed for DNA isolation according to the manufacturer’s instructions. The amount of DNA was quantified and measured photometrically using a NanoDrop UV–Vis spectrometer (NanoDrop One™, Thermo Fisher Scientific).

### Biochemical analysis of dECM

To measure the sulfated glycosaminoglycan (sGAG) content within the decellularized scaffolds, Blyscan sGAG quantitative dye-binding assay kit (Blyscan™, Carrickfergus, United Kingdom) was used according to the manufacturer’s instructions.

The amount of total collagen in dECM was quantified by colorimetric determination of hydroxyproline residues using the QuickZyme Total Collagen Assay (QuickZyme Biosciences, Leiden, The Netherlands).

### Proteomic analysis of the mice liver and kidney matrisome

Proteomic analysis was performed on ten decellularized mice liver scaffolds and three pairs of decellularized mice kidney scaffolds. In order to obtain sufficient tissue material, the samples from the simultaneously decellularized kidney pair were analyzed together. One third of each decellularized mice liver and kidney pair was prepared for shotgun proteomics using the filter-aided sample preparation (FASP) method [23]. In addition, two technical replicates were measured for five decellularized mice liver scaffolds. In brief, proteins were extracted from decellularized mice liver and kidney scaffolds by using 4% CHAPS in chilled tris-buffer (pH 7.5, 50 mM tris-base, 50 mM potassium chloride, 20% glycerol, all from Sigma-Aldrich, St. Louis, Mo, USA) supplemented with the EDTA-free protease inhibitor cocktail cOmplete™ (Roche, Mannheim, Germany). All following steps were performed on ice according to a previously published protocol by *Daneshgar *et al*.* [20]*.* Samples were mixed with 200 µl of 8 M urea in 0.1 mM Tris–HCl (pH 8.5, Sigma-Aldrich, St. Louis, Mo, USA) and incubated for 10 min at room temperature. For on-filter digestion of protein extracts using trypsin (20 µg sequencing grade modified porcine trypsin from Promega Corporation, WI, USA in 800 µl 50 mM ammonium bicarbonate from Sigma-Aldrich, St. Louis, Mo, USA), samples were transferred to Amicon Ultra membrane filter units (10 kDa molecular weight limit, Merck Chemicals GmbH) and incubated at 37°C overnight. Samples were desalted using ZipTip C18 resins (Merck Chemicals GmbH) according to the manufacturer’s specifications. Samples were measured by injecting 2 µl of the eluate into a Dionex Ultimate 3000 NanoHPLC (Thermo Fisher Scientific) coupled to an electrospray ionization quadrupole time-of-flight mass spectrometer (Impact II, Bruker Daltonic GmbH, Bremen, Germany). For the HPLC gradient that was utilized, we separated 1 µl of each peptide extract using a 2–44% acetonitrile gradient in 0.1% formic acid at a flow rate of 400 nl/min for 90 min. For de-novo sequencing, the precursor mass tolerance has been set for parent mass error tolerance to 20.0 ppm, and for fragment mass error tolerance to 0.05 Da. A monoisotopic precursor mass search type was selected, allowing for a maximum of one unique peptide and three missed cleavages. Variable oxidation, deamination, and N-terminal acetylation modifications were specified, with a significance threshold of p < 0.05. For quantification, we employed a label-free LFQ method with a feature-based LFQ with a mass error tolerance set at 10.0 ppm. Additionally, the retention time shift tolerance was set to 1.0 min, and the false discovery rate threshold was established at 1%. An analytical C18 column (Acclaim PepMap RSCL column 100 A, 75m id 150 mm, 2m particle size, Thermo Fisher Scientific) and a C18 trap column (C18, 5µm, 100 A, 300 µm i.d. x 5mm, Thermo Fisher Scientific) were used. Using the PEAKS studio database search engine (version 7.5 Bioinformatics Solutions, Waterloo, Canada) for protein identification and quantification, peak lists were searched against the murine UniProt database.

The identified proteins were categorized into different divisions of the matrisome by using *MatrisomeDB* [24, 25]*.* According to the categorization approach introduced by *Naba *et al*.* [1, 14], the identified matrisome proteins are categorized into six different groups: collagens, ECM glycoproteins, proteoglycans, ECM-affiliated proteins, ECM-regulators, and secreted factors. All mass spectrometry proteomics data have been deposited to the ProteomeXchange Consortium via the PRIDE [26] partner repository with the dataset identifier PXD049189.

### Statistical analysis

Statistical analysis was performed by using GraphPad Prism for Windows (Version 5.0, GraphPad Software Inc., La Jolla, CA, USA). Microsoft Excel (version 16.67, Microsoft, Redmond, WA 98052, USA) was used for data management. The Mann–Whitney-U test was used to compare two non-parametric variables. Quantitative results are presented as mean ± standard deviation (SD). *P* < 0.05 was defined as statistically significant.

## Results

### Fabrication of decellularized mice liver and kidney dECM scaffolds

**Fig. 1:**
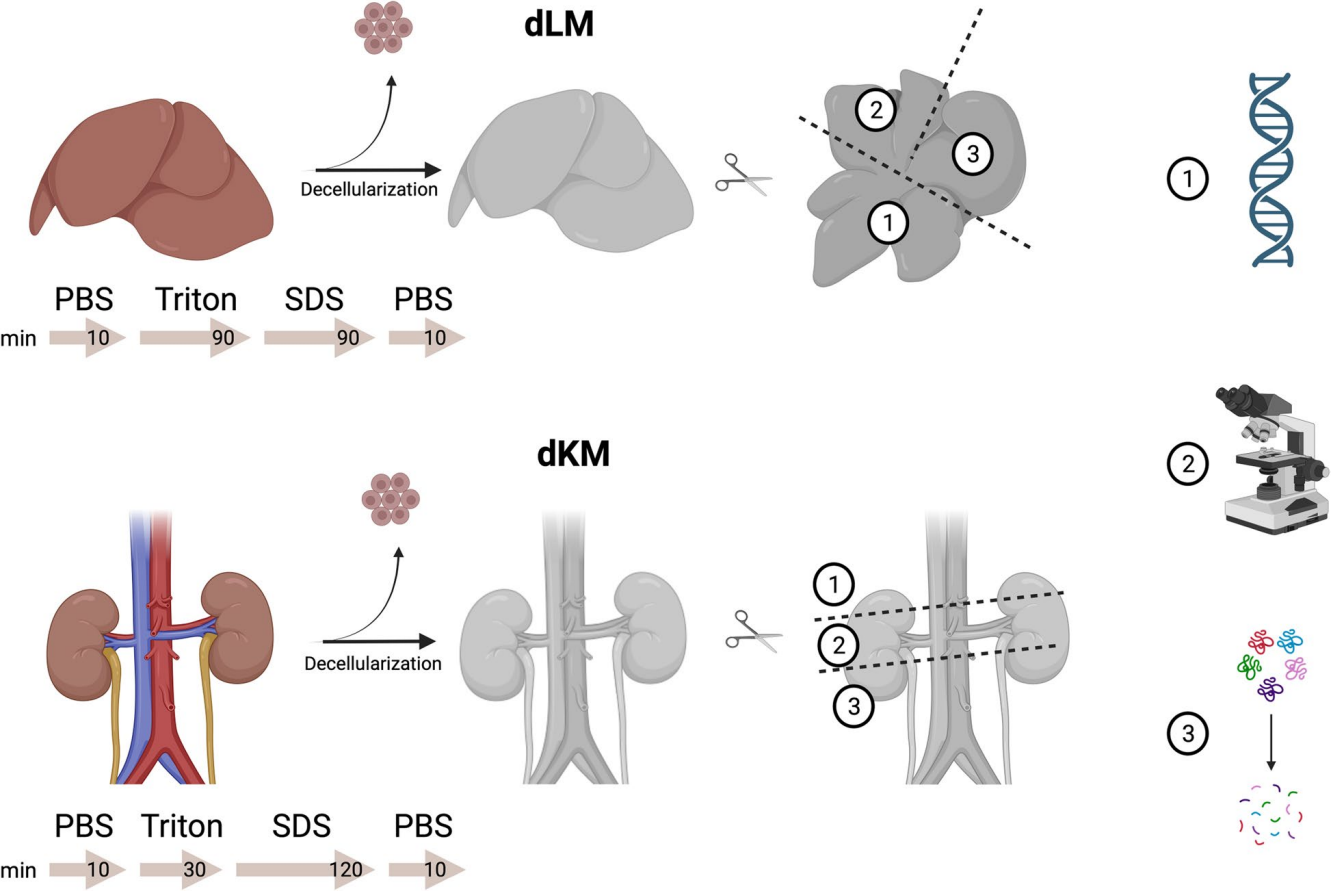
Summary of the experimental procedure for obtaining decellularized liver matrices (dLM) and decellularized kidney matrices (dKM). After decellularization, the organs were dissected and used for biochemical (1), histological (2), and proteomic (3) analysis. The figure was created with BioRender.com

In this study, we investigated the proteomic composition of decellularized mice liver and kidney extracellular matrices in combination with a novel and efficient decellularization procedure. The usage of shotgun proteomics to compare dECM scaffolds is of essential significance to understand the interaction of cells with their matrices. Ten harvested mice livers and eight mice kidneys (equivalent to four pairs) were processed into acellular dECM scaffolds by perfusion decellularization (Fig. 1). In addition, we stored ten native livers and ten native kidneys as controls.

**Fig. 2:**
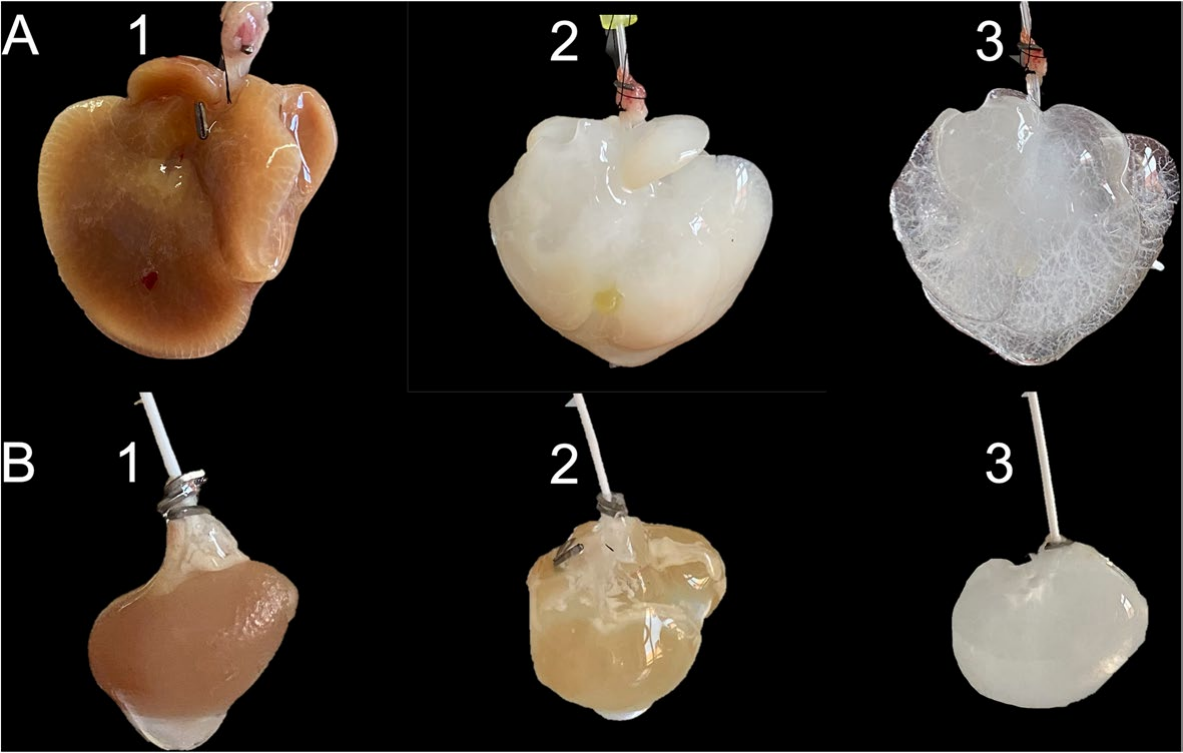
Macroscopic observations during **A** liver and **B** kidney decellularization. (1) after perfusion with PBS, (2) after perfusion with 1% Triton X-100, (3) after perfusion with 1% SDS. Initially both organs appeared in a brown color. After perfusion with Triton X-100, the liver already looked white, while the kidney was still slightly brownish. After completion of decellularization, both organs were white and translucent with a detectable vascular structure

During liver decellularization, macroscopic changes began to occur shortly after the start of perfusion with Triton X-100 (Fig. 2A). After 90 min of perfusion, the liver appeared in a whitish and non-translucent color with additional brown spots at the bottom. After completion of the decellularization process and perfusion with SDS, the organ appeared translucent while the vascular architecture could be traced to the edges of the organ. Macroscopically, the microarchitecture of the organ, including the vascular network, appeared to be intact.

During kidney decellularization, the organ appeared brownish rather than white after 30 min of perfusion with Triton X-100 (Fig. 2B). The mice kidneys became translucent under perfusion with SDS. Macroscopically, decellularization gradually progressed from the cortex toward the renal medulla. Finally, the kidneys appeared white and translucent, and the vascular network and matrix structure could also be identified.

**Fig. 3:**
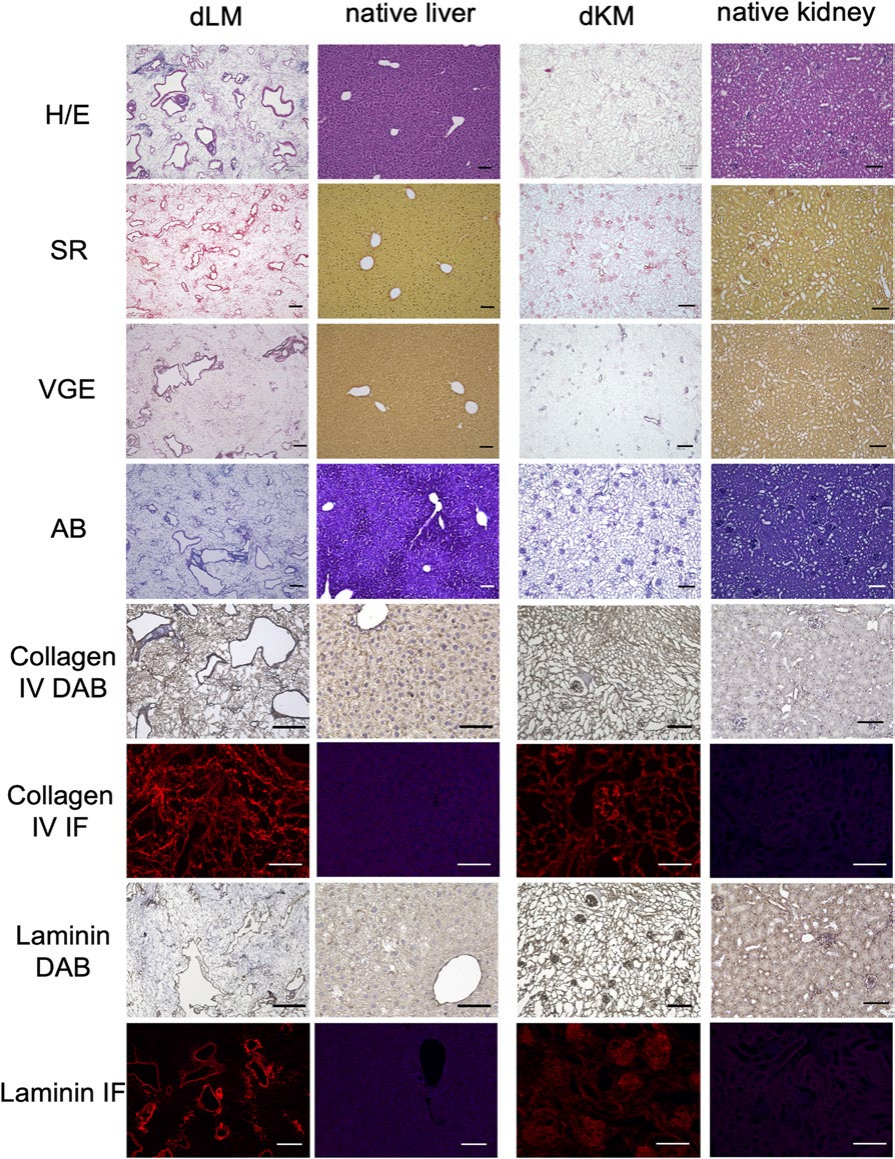
Histological and immunohistochemical evaluation of decellularized liver matrices (dLM) and decellularized kidney matrices (dKM) compared to native tissues. Stainings were performed with Hematoxylin and Eosin (H/E), Siriusred (SR), Elastica-van-Gieson (VGE) and Alcianblue (AB). For immunohistochemical analysis, DAB detection and immunofluorescence (IF) were used to visualize collagen IV and laminin expression. Overall, cells were no longer present in the decellularized samples, and the scaffold of the extracellular matrix clearly visible. No major damage to the matrix was observed. The scale bars are equal to 100 µm

Histological analysis was performed to microscopically evaluate the success of decellularization in terms of absence of cellular material and preserving the microstructure of the ECM (Fig. 3). Native tissue samples were run parallel as a control for all stainings performed. First, hematoxylin and eosin (HE) staining was conducted, which showed the removal of cellular material in both liver and kidney dECM scaffolds. The structure of the liver matrices and the architecture of the vessels were intact without any visible major destruction. In decellularized kidney scaffolds, the intact structure of the glomeruli, vessels, and tubular system was confirmed. To visualize collagen I and III fibers, Picro-Sirius red staining was performed (Fig. 3). In decellularized liver scaffolds, stained collagen fibers were seen throughout the whole tissue network, perivascular, and within vascular structures forming large parts of the ECM. In the kidney matrices, collagen fibers were visible throughout the entire matrix. To dye elastic fibers, samples were subjected to Elastica-van-Gieson staining (Fig. 3). In decellularized liver and kidney scaffolds, the elastic fibers were mainly seen in the perivascular area and were less represented in the interstitial space. To visualize sulfated glycosaminoglycans (sGAGs) and mucopolysaccharides, Alcianblue-PAS staining was performed which presented sGAGs throughout the entire tissue sections for both liver and kidney dECM scaffolds (Fig. 3). In the kidney dECM, sGAGs were located especially in the glomeruli in a nest-like arrangement.

Immunohistochemical staining for collagen IV and laminin was performed to visualize the basement membrane (Fig. 3). Both proteins play a crucial role in contributing to the structure of the matrix [27, 28]. Moreover, they are a major component of the glomerular basement membrane of the kidney [29]. In liver dECM, collagen IV was ubiquitously distributed as observed in the native tissue. On the contrary, laminin was detected particularly around the vascular network of the liver dECM and the native liver tissue. This underscores the function of laminin in the formation of the vascular basement membrane and its interaction with the endothelium [30]. In kidney dECM scaffolds, laminin was particularly present in and around the glomeruli as part of the glomerular basement membrane (Fig. 3). These findings were visible in DAB visualization and immunofluorescence detection.

**Fig. 4:**
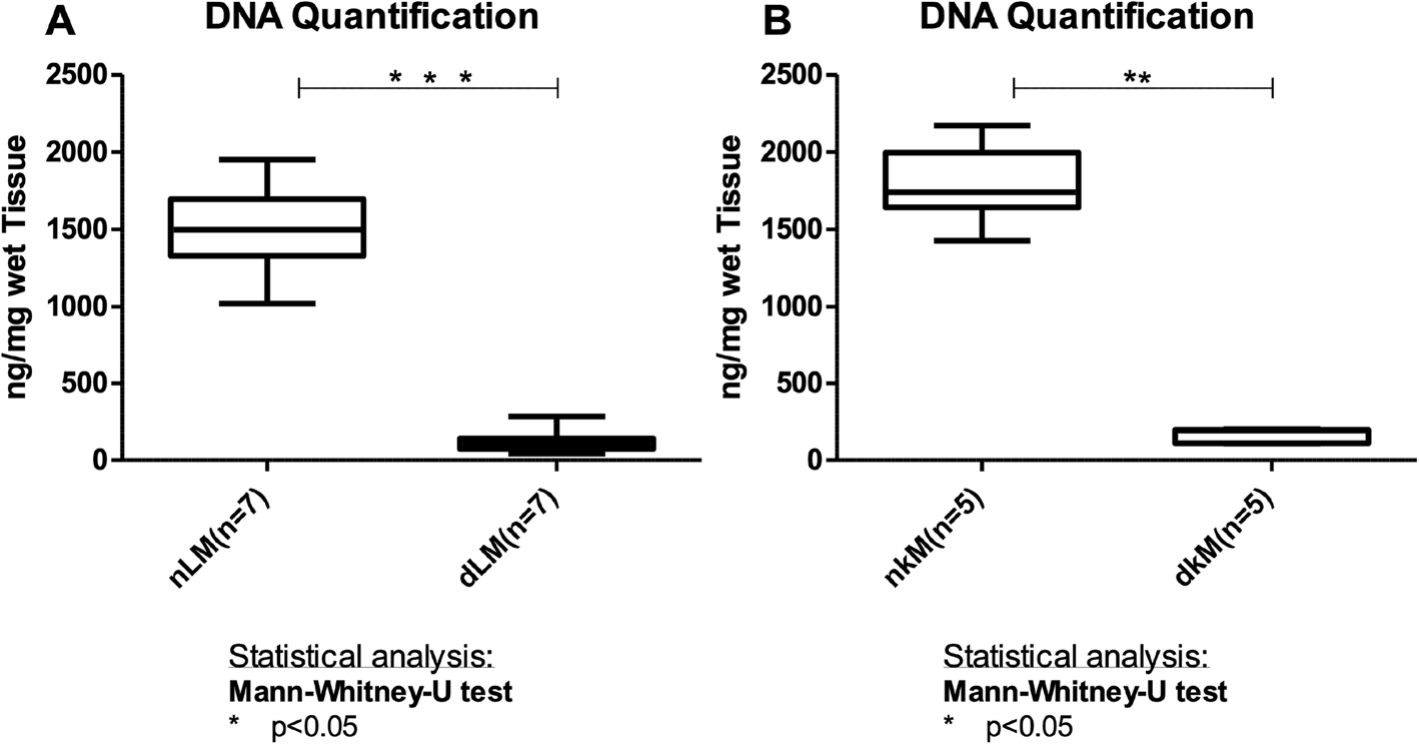
DNA content of decellularized liver matrices (dLM) and decellularized kidney matrices (dKM). **A** Quantification of the DNA content in dLM (*n* = 7) and **B** dKM (*n* = 5) compared to native tissue (nLM and nKM). A statistically significant decrease in DNA content was observed in both tissues (dLM: 126.55 ± 76.81 ng/mg, nLM: 1512.23 ± 302.86 ng/mg (*** *p* < 0.0001) dKM: 161.96 ± 46.76 ng/mg, nKM 1786.78 ± 249.47 ng/mg, ** *p* < 0.005)

DNA isolation and quantification was performed to determine residual DNA in mouse liver and kidney dECM scaffolds (Fig. 4). A significant decrease in DNA content was observed after liver decellularization with a mean ± SD of 126.55 ± 76.81 ng/mg DNA compared to a mean ± SD of 1512.23 ± 302.86 ng/mg DNA detected in native liver samples (*p* < 0.05, Fig. 4A). A residual DNA content with a mean ± SD of 161.96 ± 46.76 ng/mg was found in kidney dECM scaffolds (Fig. 4B). This represented a statistically significant decrease compared to native kidney tissue with a total DNA amount having a mean ± SD of 1786.78 ± 249.47 ng/mg (*p* < 0.05).

**Fig. 5:**
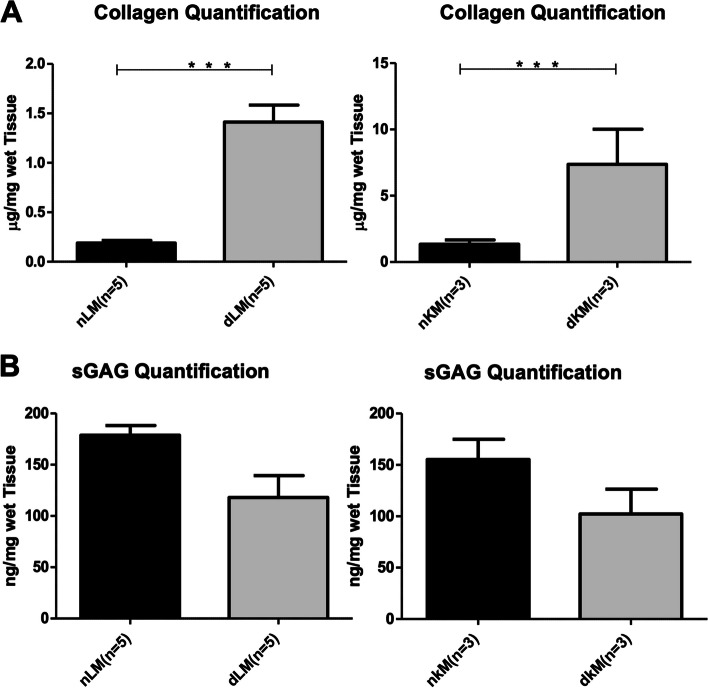
Biochemical analysis of decellularized matrices. Collagen (**A**) and sGAG (**B**) content was quantified for decellularized liver (dLM) and kidney (dKM) matrices compared to native tissues (nLM and nKM). The amount of collagen in µg per mg of wet tissue was significantly higher in the decellularized tissue of both organs (dLM: 1.82 ± 1.84 µg/mg, nLM: 0.19 ± 0.09 µg/ mg (*** *p* < 0.0001); dKM: 7.37 ± 8.37 µg/mg, nKM: 1.34 ± 1.06 µg/mg (*** *p* < 0.0001)). The sGAG content in ng per mg of wet tissue showed no statistically significant difference between the decellularized and native samples (dLM: 92.60 ± 65.37 ng/mg, nLM: 171.92 ± 50.65 ng/mg (*p* > 0.05); dKM: 102.30 ± 80.05 ng/mg, nKM: 155.30 ± 73.24 ng/mg (*p* > 0.05)). Nevertheless, a trend in favor of the native tissue was observed

To further investigate the preservation of essential ECM components after decellularization, sGAG content and collagen levels were quantified (Fig. 5). A significant difference in collagen content was observed for both the kidney and the liver before and after decellularization (Fig. 5A). This can be linked to the “enrichment” of ECM components after decellularization due to the loss of cellular material (decellularized liver ECM (dLM): 1.82 ± 1.84 µg/mg, native liver tissue (nLM): 0.19 ± 0.09 µg/mg (*p* < 0.05); decellularized kidney ECM (dKM): 7.37 ± 8.37 µg/mg, native kidney tissue (nKM): 1.34 ± 1.06 µg/mg (*p* < 0.05)). Quantification of sGAG content in mice livers and kidneys before and after decellularization presented a slightly higher amount of sGAGs in the native tissue compared to the decellularized scaffolds. However, no statistically significant difference was detected in the measurement of the sGAG content after decellularization of mice livers and kidneys as shown in Fig. 5B (dLM: 92.60 ± 65.37 ng/mg, nLM: 171.92 ± 50.65 ng/mg (*p* > 0.05); dKM: 102.30 ± 80.05 ng/mg, nKM: 155.30 ± 73.24 ng/mg (*p* > 0.05)).

Overall, the results of the histological and biochemical examination confirm that the decellularization was successful and that the majority of the cellular components were removed while the structure of the ECM was largely preserved.

### Proteomic analysis of mice liver dECM scaffolds

**Fig. 6:**
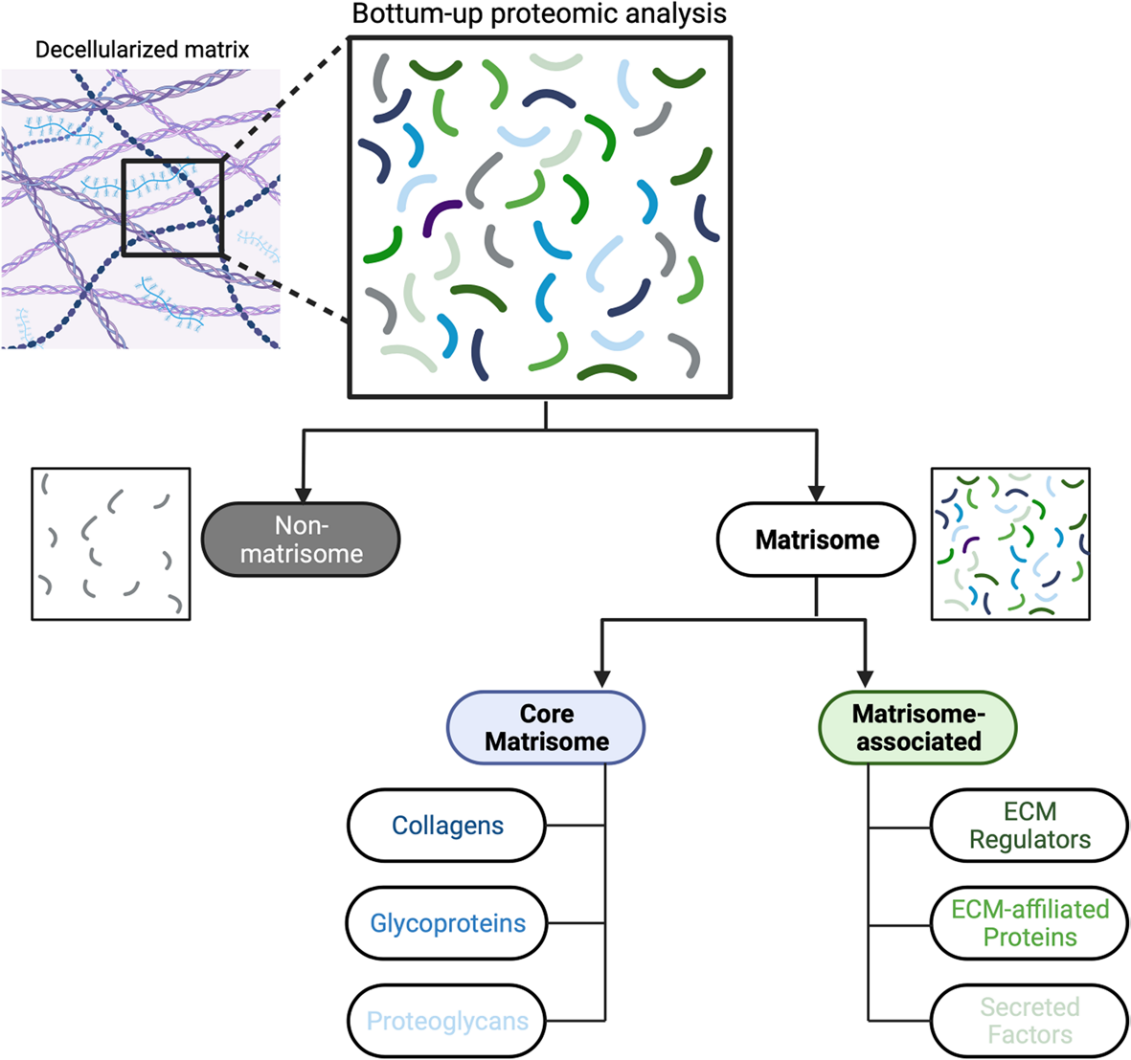
Flowchart of the protein classification of decellularized extracellular matrices after proteomic analysis according to MatrisomeDB. First, matrisome and non-matrisome proteins were distinguished. Then, the matrisome proteins were further divided into the core matrisome division with collagens, glycoproteins, and proteoglycans, and the matrisome-associated division with ECM-regulators, ECM-affiliated proteins, and secreted factors. This figure was created with BioRender.com

In order to gain an in-depth insight into the ECM proteome of the mouse liver dECM, proteomic analysis of ten decellularized mice liver dECM scaffolds compromising two technical replicates from five decellularized mice liver dECM scaffolds was conducted and revealed an average of 959.7 ± 152.9 proteins per liver dECM scaffold. The identified proteins were further categorized into the different divisions of the matrisome using *MatrisomeDB* [24, 25] (Fig. 6).

The matrisome can be classified into the core matrisome, which consists of collagens, proteoglycans, and glycoproteins, and the matrisome-associated division which contains secreted factors, ECM-regulators, and ECM-affiliated proteins. By employing the matrisome categorization approach introduced by *Naba *et al. [1], a total of 112 different matrisome proteins were found across all liver samples, of which 66 were categorized as core matrisome proteins (58.93%) and 46 as matrisome-associated proteins (41.07%) (Supplementary Table 1).

On average, a mean number of 68.27 ± 10.03 matrisomal proteins were identified per mouse liver dECM scaffold. With respect to the spatial distribution of matrisome proteins, two technical replicates were measured for five decellularized liver dECM scaffolds taken from different parts of a liver lobe. For each pair of replicates, the concordance of the detected proteins was determined, resulting in an average concordance across the five liver dECM replicates of 93.73% for core matrisome proteins and 62.69% for matrisome-associated proteins.

**Fig. 7:**
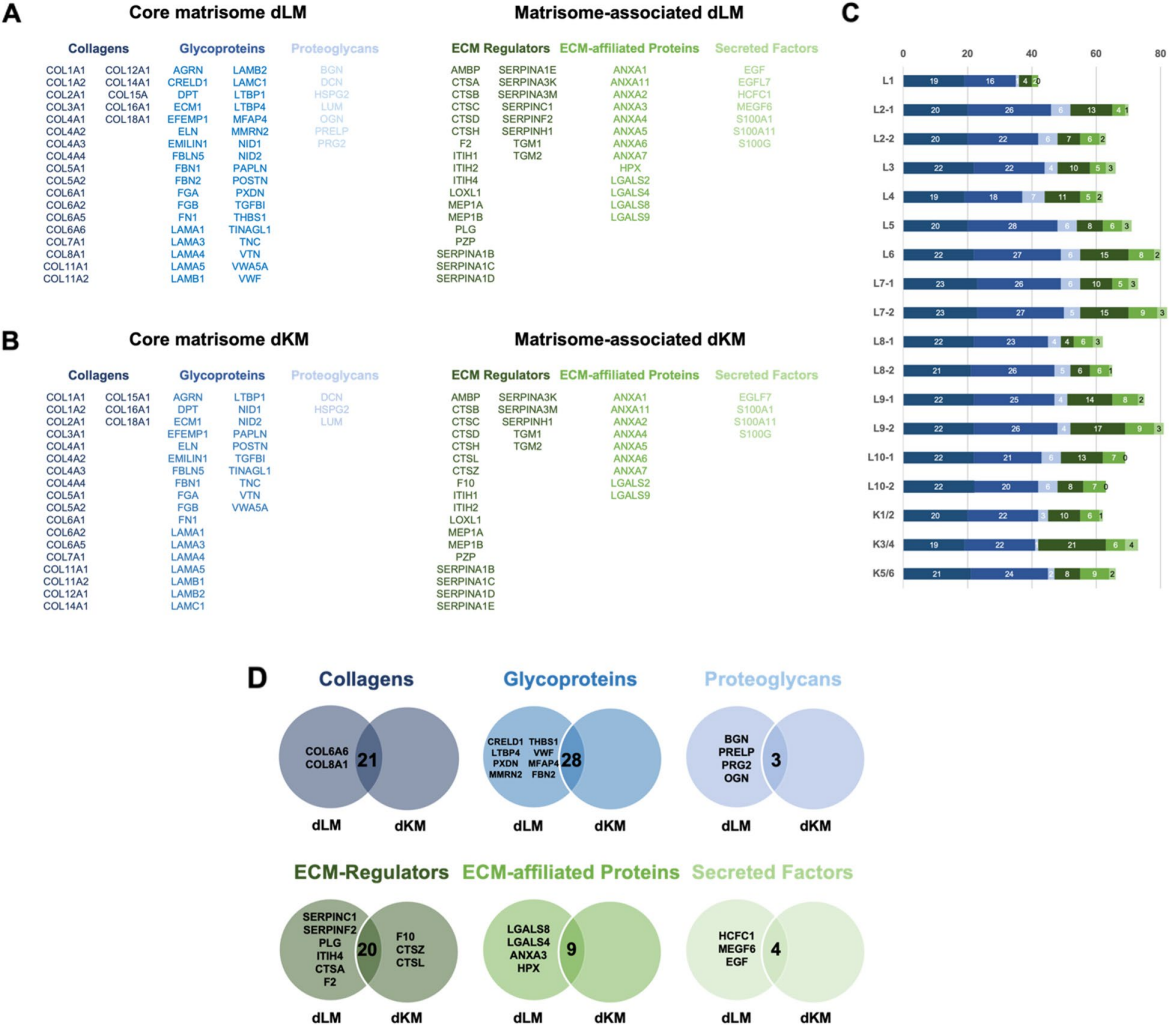
**A** Overview of matrisome proteins found in decellularized mice liver ECM (dLM) and **B** in decellularized mice kidney ECM (dKM), categorized into different divisions and subgroups of the matrisome by using MatrisomeDB. **C** Graphical representation of the subgroups according to individual samples. **D** Venn diagrams showing the differences between the matrisome of decellularized liver ECM and decellularized kidney ECM, each diagram representing a subgroup of the matrisome, with organ-specific gene codes on the corresponding side

Across all liver dECM samples, a total of 23 collagens, 36 glycoproteins and 7 proteoglycans were found corresponding to the core matrisome. Furthermore, 26 ECM-regulators, 13 ECM-affiliated proteins, and 7 secreted factors were detected in the matrisome-associated division of the liver dECM samples (Fig. 7A). The overall number of matrisome proteins detected in each sample varied from 42 to 82 (Fig. 7C). In total, 34 of the 112 proteins were detected in all decellularized liver samples. Of these, 31 belonged to the core matrisome and 3 to the matrisome-associated division. 52 proteins were found in at least two thirds of the samples.

Notably, the number of identified core matrisome proteins varied very little between different samples (collagens: 21.27 ± 1.33, glycoproteins: 23.53 ± 3.6, proteoglycans: 5.07 ± 1.49). A particularly high level of redundancy was found in the collagen group. In contrast, more variation was observed in the matrisome-associated division (ECM-regulators: 10.33 ± 4.11, ECM-affiliated proteins: 6.2 ± 1.9, secreted factors: 1.87 ± 1.19). Thus, the overall variation in the number of matrisome proteins identified is mainly linked to the variation in the number of matrisome-associated proteins detected.

### Proteomic analysis of mice kidney dECM scaffolds

Proteomic analysis of three pairs of mice kidney dECM scaffolds revealed an average of 1190 ± 338 proteins per kidney dECM scaffold. We had to omit technical replicates for proteomic analysis due to the small amount of material in favor of histological and biochemical analysis. Identified proteins were further categorized into the different divisions of the matrisome using *MatrisomeDB* [24, 25] as described above (Fig. 6). A total of 88 different matrisome proteins were found across all three pairs of mice kidney dECM scaffolds. Of these, 52 proteins corresponded to the core matrisome (59.1%) and 36 to the matrisome-associated division (40.9%) (Supplementary Table 1).

Resembling the distribution of proteins in different matrisome categories in mice liver dECM scaffolds, a total of 21 collagens, 28 glycoproteins, 3 proteoglycans, 23 ECM-regulators, 9 ECM-affiliated proteins and 3 secreted factors were identified across all kidney dECM scaffolds (Fig. 7B). On average, 67 ± 5.57 matrisome proteins were detected per kidney dECM sample. The overall number of matrisome proteins ranged from 62 to 73 proteins (Fig. 7C).

There was a high stability and redundancy in the amount of core matrisome proteins (collagens: 20 ± 1, proteoglycans: 2 ± 1, glycoproteins: 22.67 ± 1.15), whereas the number of matrisome-associated proteins was more variable (ECM-regulators: 13 ± 7, ECM-affiliated: 7 ± 1.73, secreted factors: 2.33 ± 1.53). 48 of the 88 matrisome proteins were detected in all decellularized kidney samples, while 65 of the proteins were found in at least 2 out of 3 kidney pairs. The variation in the total number was mainly due to the variation in the number of matrisome-associated proteins, as we observed for the liver dECM scaffolds. The number of core matrisome proteins in kidney dECM scaffolds varied from 45 to 47, while the number of matrisome-associated proteins ranged from 17 to 31.

### Comparison of mice liver and kidney dECM scaffolds

For each division of matrisome proteins, a Venn diagram was generated to highlight differences and similarities between mice liver and kidney dECM scaffolds (Fig. 7D). For our analyses, we focused on comparing the core matrisome, given its relative stability in our studies.

The collagen division revealed 21 overlaps and 2 additional unique proteins for the liver dECM scaffolds. Collagen type VI alpha-6 (Col6a6) was found in 14 of 15 liver dECM samples, whereas it was absent in kidney dECM scaffolds. This collagen variant was only identified in 2008 and its tissue-specific roles have not been clearly defined. It is known to occur mainly in the interstitial matrix and to show a close association with the basement membrane. Its occurrence appears to be restricted in distribution, but with highly differentiated functions [31–33].

For glycoproteins, 28 matches were found between the mouse liver dECM and kidney dECM proteome with another eight whose expression was restricted to the liver. Latent-transforming growth factor beta-binding protein 4 (Ltbp4) has been identified as one of these liver-specific proteins. It plays an important role in the activation of transforming growth factor ß (TGF-ß), which acts as a multifunctional growth factor [34]. Ltbp4 deficiency leads to pulmonary emphysema, cardiomyopathy, colorectal cancer, and profound defects in the elastic fiber structure of the ECM [35]. Another glycoprotein of potential interest was fibrillin-2 (Fbn2). It has both structural functions in maintaining the tissue integrity of the ECM and regulatory functions through interactions with elastic fibers and growth factors such as TGF-ß [36].

**Fig. 8:**
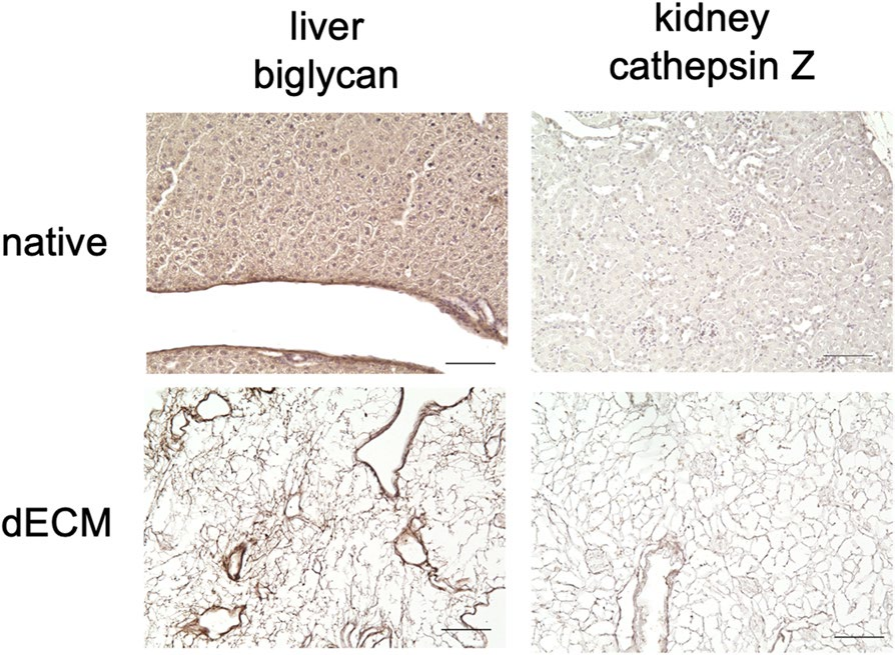
Immunohistochemical staining on decellularized and native liver tissues for biglycan, and on decellularized and native kidney tissues for cathepsin Z. The specific detection of biglycan was found to be ubiquitous in the ECM of liver tissues, while cathepsin Z staining was observed in a spot-like pattern in the ECM of kidney tissues. The scale bar equals to 100 µm

In addition, four proteoglycans have been found specifically in the liver. One of these proteoglycans was biglycan (Bgn), which is involved in collagen fiber assembly [37]. Studies have shown that increased biglycan levels are associated with enhanced proliferation, motility, tumorigenesis, and liver metastasis of colorectal tumors [38]. To confirm the presence of biglycan in the decellularized liver, we additionally performed immunohistochemical staining for native and decellularized liver tissue (Fig. 8). The staining revealed the presence of biglycan in the intercellular space of the ECM in both tissues.

Although it does not fall within the division of the core matrisome, it is noteworthy that cathepsin Z (CtsZ) was detected in 2 out of 3 kidney pairs, whereas it was not found in any of the liver samples. Cathepsin Z is an exopeptidase with monocarboxypeptidase activity and belongs to the family of cysteine cathepsin proteases [39]. It also interacts with several integrins during normal homeostasis and plays an important role in cell signaling [40]. *Akkari *et al*.* [41] investigated cancer-promoting functions of cathepsin Z and found that it had a regulatory function and influenced the development of a favorable tumor microenvironment. An immunohistochemical control was conducted for the kidney-specific protein cathepsin Z (Fig. 8). The staining of the native tissue showed a weak but clear brownish staining in spots, which may be attributed to partial distribution of cathepsin Z as an enzyme. Similarly, spot-like structures were visible in the decellularized tissue.

To conclude, the bottom-up proteomic analysis allowed us to identify several matrisome proteins, indicating a mostly stable core matrisome composition. Moreover, this confirms the efficacy of our decellularization approach in preserving ECM structures.

## Discussion

This study presents an innovative approach to characterize the proteomic composition of decellularized mice liver and kidney extracellular matrices. To the best of our knowledge, this study is the first to present an in-depth analysis of the ECM proteome of both organs using perfusion-based whole organ decellularization. Understanding and interpreting the structural and biochemical crosstalk between cells and their microenvironment in the emergence of diseases requires elucidating the complex proteomic composition of natural matrices that act as scaffold and interaction medium between both compartments. The extracellular matrix plays a crucial role in cellular signaling pathways through its interaction with cell adhesion receptors, determining processes such as cell growth, survival, migration, and differentiation [3, 4, 42]. To investigate cell–matrix interactions, a more detailed analysis of the matrisome is one important prerequisite. The decellularization method offers a potent tool for examining the composition of the ECM and opens up the possibility to investigate a wide range of physiological and pathological processes through recellularization of different decellularized organ matrices with different cell types or lines [43, 44]. Due to the high molecular weight, insolubility and strong cross-linking of proteins, a precise analysis of the composition of the matrisome is challenging [45]. Therefore, proteomic analysis has become an essential technique to shed light on proteomic variation and turnover in complex biological scaffolds [4, 42]. Two frequently used methods in proteomic studies are top-down and bottom-up proteomics. Top-down proteomics involves directly separating and analyzing intact proteins with LC–MS/MS to both characterize and quantify proteins. On the other hand, bottom-up proteomics begins with enzymatically digesting proteins into peptides before analyzing and identifying them [18, 46]. Especially shotgun proteomic analysis can provide critical information on even low abundance matrisome proteins that will contribute to our overall understanding of the biological interactions that occur in engineered biological scaffolds. In our study we employed a bottom-up proteomic approach and chose the filter-aided sample preparation (FASP) technique. This method has been shown to provide in depth-coverage and to remove residual SDS from the decellularized scaffolds during the process [23, 47]. Although the decellularization protocols used for each organ were identical, the amount and type of matrisome-associated proteins varied, while the core matrisome remained largely stable. This may indicate non-specific changes in the ECM caused by decellularization. Although SDS is known to be effective in removing cells, prolonged detergent incubation may cause degradation and elimination of matrisomal proteins and alteration of the matrix [48, 49].

An important step in the proteomic investigation of the matrisome is the matrisome enrichment process, which aims to remove cellular components from the tissue. Currently, numerous technical approaches exist for matrisome enrichment procedures, but it remains unclear which one is the most suitable [50, 51]. In general, ECM enrichment strategies can be categorized into those using decellularization and those sequentially extracting ECM from native or tissue homologues [52]. Decellularization-based matrisome enrichment methods have been proven particularly effective in preserving the core matrisome as shown in previous studies [45]. For example, *Calle *et al*.* [53] have demonstrated the preservation of collagens and laminins by decellularizing murine lungs and comparing them to native tissue. *Leng *et al*.* [54] have shown that after decellularizing porcine skin a significant enrichment of collagens and glycoproteins occurred. *Daneshgar *et al*.* [20] reported that the decellularization process primarily affects the division of matrisome-associated proteins, while preserving the core matrisomal proteins. The stability of the core matrisome during decellularization and the variability of matrisome-associated proteins align with the findings of our study.

Instead of decellularization-based approaches, sequential ECM extraction methods are used for matrisome enrichment, such as after tissue homogenization. This method was also utilized by *Naba *et al*.* [1] and has been successfully applied to other tissues such as the liver [55], pancreas [56], and vascular tissues [57].

Both methods have limitations and advantages that depend on tissue type and analysis focus.

For our study, we decided to perform whole organ perfusion decellularization, which has not been combined with proteomic analysis in this form in any other study. This technique has the advantage of preserving the structural integrity, three-dimensional architecture, and vascular network of the entire organ and opens up the later recellularization of the tissue. Further, we conducted a comprehensive proteomic analysis of decellularized mice liver and kidney dECM scaffolds, which identified numerous matrisomal and non-matrisomal proteins, highlighting the complexity of the matrix structure.

In previous studies on the mouse liver matrisome, for example, *Yuzhalin et. al* [58] were able to assign 140 matrisome proteins to the mouse liver, of which 50 belonged to glycoproteins, 31 to ECM-regulators, 24 to collagens, 18 to ECM-affiliated proteins, 9 to proteoglycans and 8 to secreted factors. This is consistent with our findings that most of the detected proteins belong to the glycoproteins or ECM-regulators, whereas proteoglycans or secreted factors were identified less. Furthermore, four different matrisome enrichment methods were compared by *Krasny *et al*.* [50]. Depending on the method used, up to 40 different matrisome proteins were detected. They also found that there was a loss of matrisome-associated proteins, independent of the enrichment process. This agrees with our observation that the number and type of matrisome-associated proteins were much more variable than the core matrisome proteins. One possible explanation for this finding is that many of the matrisome-associated proteins, especially the secreted factors, are soluble and thus lost during the decellularization process.

There are also previous studies in which the matrisome of the mouse kidney has been investigated [51, 59–61]. In these studies, the total number of matrisome proteins ranged from 79 to 173, with an average of 126 different proteins detected, compared to our result of 88 proteins. The largest proportions were either glycoproteins or ECM-regulators, and the fewest were proteoglycans or secreted factors, which is consistent with our findings.

In detail, in the other four studies a total of 25 different collagens were found, 20 of which were also detectable in our samples. In addition, we detected collagen type VII alpha 1 (COL7A1), which was not found in any of the other groups. COL7A1 is part of the basement membrane and acts primarily as an anchoring fibril between the basement membrane and the proximal cells of squamous epithelia [62]. Furthermore, we were able to detect 28 different glycoproteins, all of which were also found by the other groups. Overall, relatively few proteoglycans were found, between six to ten per study. Our three detected proteoglycans had also been identified in the other four studies. 23 different ECM-regulators were found in our study, 21 of which were also described in previous studies. In addition, we detected two novel ECM-regulators: coagulation factor X (F10) and alpha-2-macroglobulin-like (PZP). While F10 is an enzyme and part of the coagulation cascade [63], PZP is discussed as both a protease inhibitor and a T-cell modulator, but the exact function remains to be determined [64]. Eight proteins of our samples were classified as ECM-affiliated proteins, which were also found in the other studies. In addition, galectin-2 (LGALS2) was detectable in our study. Galectin-2 belongs to the ß-galactoside-binding proteins and is involved in cell surface receptor binding and in the regulation of several physiological and pathological conditions such as epithelial layer integrity, inflammation, immune response, and apoptosis [65, 66]. Among the secreted factors, the four proteins we found were also present in the other groups. However, there was little similarity between the described proteomes. Per study, two to ten secreted factors were found, but only one was found in all studies, including ours: S100 calcium-binding protein A11 (S100A11). Overall, our study of the mouse kidney matrisome yielded results that align with previous research findings. While the total protein count was relatively low, we were able to discover new matrisome proteins not detected in prior studies. Our findings will aid in deciphering and understanding the proteomic composition of decellularized mice kidneys, particularly given that the ECM comprises the interstitial connective tissue and glomerular basement membrane, which play a critical role in the organ's physiology [67].

Furthermore, we present a proof-of-concept by comparing matrisomes from decellularized mice livers and kidneys. We identified proteins that were specific for the liver dECM scaffolds, but not present in the kidney dECM scaffolds. However, we recognize that our study is limited by differences in sample sizes, which may have influenced the results and resulted in only a small number of kidney-specific proteins. In addition, we are aware of the spatial distribution of the ECM within an organ. For this reason, we examined technical replicates for the liver, although this cannot represent the spatial distribution in its entirety. Here, matrix-assisted laser desorption/ionization imaging mass spectrometry (MALDI IMS) imaging is a promising and powerful approach for preserving the spatial distribution and analyzing biomolecules within a tissue with high sensitivity and specificity. This approach provides information on both the relative abundances and spatial arrangement of proteins within the matrix [68–70].

The use of dECMs is a well-established technology that originated mainly in the fields of tissue engineering and regenerative medicine [71]. Bioactive materials derived from extracellular matrices of natural origin have emerged as promising novel materials, enabling a wide use in various tissue engineering and other research approaches [43, 72, 73]. Preserving the native tissue morphology enables research on the influence of cells on the intricate host ECM and the interactions of distinct cell populations within an organ scaffold. Decellularized ECM biomaterials have been repopulated with cells and used to replace organs for therapeutic purposes in end-stage organ failure, including heart [74], liver [75], lung [76], and kidney [77]. They are also used in the study of cell growth and function [78], in drug testing [79], and in the modeling of diseases, including cancer [80, 81]. Decellularized scaffolds have proven to be particularly useful in the field of oncological research due to the critical role of the ECM in creating a specific tumor microenvironment [82]. A major obstacle in cancer research is the restricted availability of models to investigate these tumor-stroma and associated tumor-ECM interactions where the concept of organ de- and recellularization may aid in overcoming by ex vivo mimicking in vivo conditions of real organs, tumors, or metastases. There have been studies that have analyzed the behavior of malignant cells inoculated on decellularized scaffolds [80], either by directly decellularizing tumorous tissues to analyze the pre-existing cancerous matrix [83–85] or by decellularizing healthy tissue samples to analyze tumor cell-induced alterations of the ECM after recellularization with cancer cells [86–88].

Overall, these studies indicate great potential for using decellularized organs as research platforms, especially the addition of proteomics and matrisome analysis can help identify essential proteins and structures to improve our understanding in unraveling the complex ECM composition. However, it is important to acknowledge that although decellularized organ scaffolds hold promise, there are challenges that need to be addressed. Currently, there is a lack of standardized protocols for the process of decellularization, consecutive sterilization, and recellularization. In addition, the immunogenicity of the organ scaffold presents a significant challenge, particularly for further transplantation approaches. In the context of recellularization, it is crucial to maintain scaffold integrity and achieve successful reendothelialization by utilizing different cell types including endothelial cells, stem cells, fibroblasts, and additional growth factors. Finally, it is required to translate animal findings to humans and to verify the use of human primary cells.

### Limitations

We acknowledge certain limitations when looking at our study. Firstly, cellular remnants that remain after decellularization may affect proteomic analysis. Moreover, the decellularization process may cause alteration of the ECM. As the surgical method was technically demanding, fewer kidneys than livers were decellularized, resulting in a smaller sample size. Also, as there was less kidney tissue available, we were not able to include technical replicates for this organ in our study. Furthermore, the smaller number of kidney samples analyzed makes it harder to compare the results with those of the decellularized liver scaffolds.

## Conclusion

In this study, we present a novel approach to analyze mice liver and kidney extracellular matrix scaffolds on a proteomic level to identify key components that will contribute to tissue-specific functionality. By employing effective whole organ decellularization, we created naturally-derived mice liver and kidney dECM scaffolds that can be used for various research approaches. We hereby provide information on the mice liver and kidney matrisome that will be relevant for interpreting cell–cell and cell–matrix interactions when studying tissue physiology and pathophysiology in mouse models. Bottom-up proteomic analysis revealed differences between the mice liver and kidney matrisomes that concern especially variations in the matrisome-associated division of the ECM. The results obtained underscore the relevance of employing proteomic techniques to identify variations in less represented factors and regulators within decellularized scaffolds. Furthermore, the presented workflow can provide additional cues to the scaffold fabrication process and is essential for the evaluation of biological matrices.

### Supplementary Information

**Supplementary material 1.**

**Supplementary material 2.**

## Data Availability

All mass spectrometry proteomics data have been deposited to the ProteomeXchange Consortium via the PRIDE partner repository with the dataset identifier PXD049189. To link the raw files to the sample abbreviations used in the manuscript, a metadata file was created, which can be found in Supplementary Table 2.
